# Safety and possible anti-inflammatory effect of paclitaxel associated with LDL-like nanoparticles (LDE) in patients with chronic coronary artery disease: a double-blind, placebo-controlled pilot study

**DOI:** 10.3389/fcvm.2024.1342832

**Published:** 2024-02-21

**Authors:** Lucas Lage Marinho, Fabiana Hanna Rached, Aleksandra Tiemi Morikawa, Thauany Martins Tavoni, Ana Paula Toniello Cardoso, Roberto Vitor Almeida Torres, Antonildes Nascimento Assuncao, Carlos Vicente Serrano, Cesar Higa Nomura, Raul Cavalcante Maranhão

**Affiliations:** ^1^ Lipid Metabolism Laboratory, Instituto do Coracao (InCor) Universidade de Sao Paulo, São Paulo, Brazil; ^2^ Department of Cardiopneumology, Instituto do Coracao (InCor) Universidade de Sao Paulo, São Paulo, Brazil; ^3^ Department of Radiology, Instituto do Coracao (InCor) Universidade de Sao Paulo, São Paulo, Brazil

**Keywords:** atherosclerosis, coronary artery disease, nanoparticles, paclitaxel, inflammation, interleukin-6, drug-Related side effects and adverse reactions

## Abstract

**Introduction:**

Studies in cholesterol-fed rabbits showed that anti-proliferative chemotherapeutic agents such as paclitaxel associated with solid lipid nanoparticles (LDE) have marked anti-atherosclerotic effects. In addition, association with LDE nearly abolishes paclitaxel toxicity. We investigated whether treatment with LDE-paclitaxel changes plaque progression by coronary CT angiography and is safe in patients with chronic coronary artery disease.

**Methods:**

We conducted a prospective, randomized, double-blind, placebo-controlled pilot study in patients with multi-vessel chronic coronary artery disease. Patients were randomized to receive IV infusions of LDE-paclitaxel (paclitaxel dose: 175 mg/m^2^ body surface) or LDE alone (placebo group), administered every 3 weeks for 18 weeks. All participants received guideline-directed medical therapy. Clinical and laboratory safety evaluations were made at baseline and every 3 weeks until the end of the study. Analysis of inflammatory biomarkers and coronary CTA was also performed at baseline and 4 weeks after treatment.

**Results:**

Forty patients aged 65.6 ± 8 years, 20 in LDE-paclitaxel and 20 in placebo group were enrolled. Among those, 58% had diabetes, 50% had myocardial infarction, and 91% were in use of statin and aspirin. Baseline demographics, risk factors, and laboratory results were not different between groups. In all patients, no clinical or laboratory toxicities were observed. From the baseline to the end of follow-up, there was a non-significant trend toward a decrease in IL-6 levels and hsCRP in the LDE-paclitaxel group (−16% and −28%, respectively), not observed in placebo. Regarding plaque progression analysis, variation in plaque parameter values was wide, and no difference between groups was observed.

**Conclusion:**

In patients with multivessel chronic coronary artery disease and optimized medical therapy, LDE-paclitaxel was safe and showed clues of potential benefits in reducing inflammatory biomarkers.

**Clinical Trial Registration:**

https://clinicaltrials.gov/study/NCT04148833, identifier (NCT04148833).

## Introduction

Cardiovascular diseases (CVDs) are the leading cause of death globally (1). Coronary artery disease (CAD) accounts for 8.9 million deaths globally and one-third of deaths in Brazil (2). Atherosclerosis is a multifactorial disease involving not only cholesterol accumulation in the arteries, but also genetic, environmental, metabolic, and immunologic factors (3). It is now well-established that the inflammatory process plays a crucial role in plaque formation and progression to thrombotic complications that account for the major adverse cardiovascular events (MACE) (4).

Advancements in the treatment of atherosclerotic disease over the past few decades, resulting from therapeutic interventions and lifestyle changes, have led to increased survival and a marked reduction in cardiovascular events in the population. The increasing use of statins, antiplatelet agents, and the control of key risk factors such as hypertension, diabetes mellitus (DM), and smoking cessation decisively contributed to reducing the risk of MACE. However, even with adequate control of those major risk factors, a significant percentage of individuals still experience recurrent cardiovascular events (5). In a collaborative analysis of contemporary studies, among patients receiving statins, inflammation assessed by high-sensitivity CRP was a stronger predictor of residual cardiovascular event risk than cholesterol assessed by low-density lipoprotein (LDL) (6).

In view of the key role of inflammation in atherogenesis, different anti-inflammatory strategies were proposed to mitigate the atherosclerosis burden (7). Our laboratory has been pursuing the use of chemotherapeutic agents used in cancer treatment to achieve this end, since those agents have potent antiproliferative and immunomodulating actions. The toxicity of chemotherapeutic agents was drastically reduced by using nanoparticles that resemble the lipid structure of low-density lipoproteins (LDL) as vehicles of those drugs in circulation (8, 9). The nanoparticles carrying the associated drugs are taken up by the cells of inflammatory tissues via the LDL receptor mediated endocytic pathway. Pre-clinical data showed that chemotherapeutic agents such as paclitaxel associated with solid lipid nanoparticles (LDE) had marked anti-atherosclerotic effects (10). In rabbits with atherosclerosis induced by cholesterol feeding, treatment with LDE-paclitaxel reduced the lesion area by 60%, and the intima-media ratio fourfold, while inhibiting both macrophage invasion of the intima and smooth muscle cell proliferation.

The aim of the current pilot study was to test the toxicity and safety of LDE-paclitaxel in patients with multivessel chronic coronary artery disease and to observe whether this formulation could have effects on inflammatory biomarkers and atherosclerotic plaque progression.

## Methods

### Study design and population

This was a prospective, randomized, double-blinded, single-center pilot study at the Heart Institute (InCor), University of Sao Paulo Medical School Hospital. This study aimed to assess the safety and the effect of LDE-paclitaxel therapy on coronary plaque morphology in patients with multivessel chronic coronary artery disease and optimized medical therapy. To date, there is no parallel in the literature of randomized clinical trials with LDE-paclitaxel for the treatment of patients with coronary artery disease. Sample size was calculated based on Vaidya K et al. (11), where patients treated with a low dose of colchicine exhibited an average reduction of 41% in low attenuation plaque volume (mm^3^) over 12 months. To achieve a 25% reduction in low attenuation plaque volume (mm^3^) in our study, with a power of 80% and an alpha of 5%, we estimated that at least 38 patients would be required, with 19 in each group.

**Figure 1:**
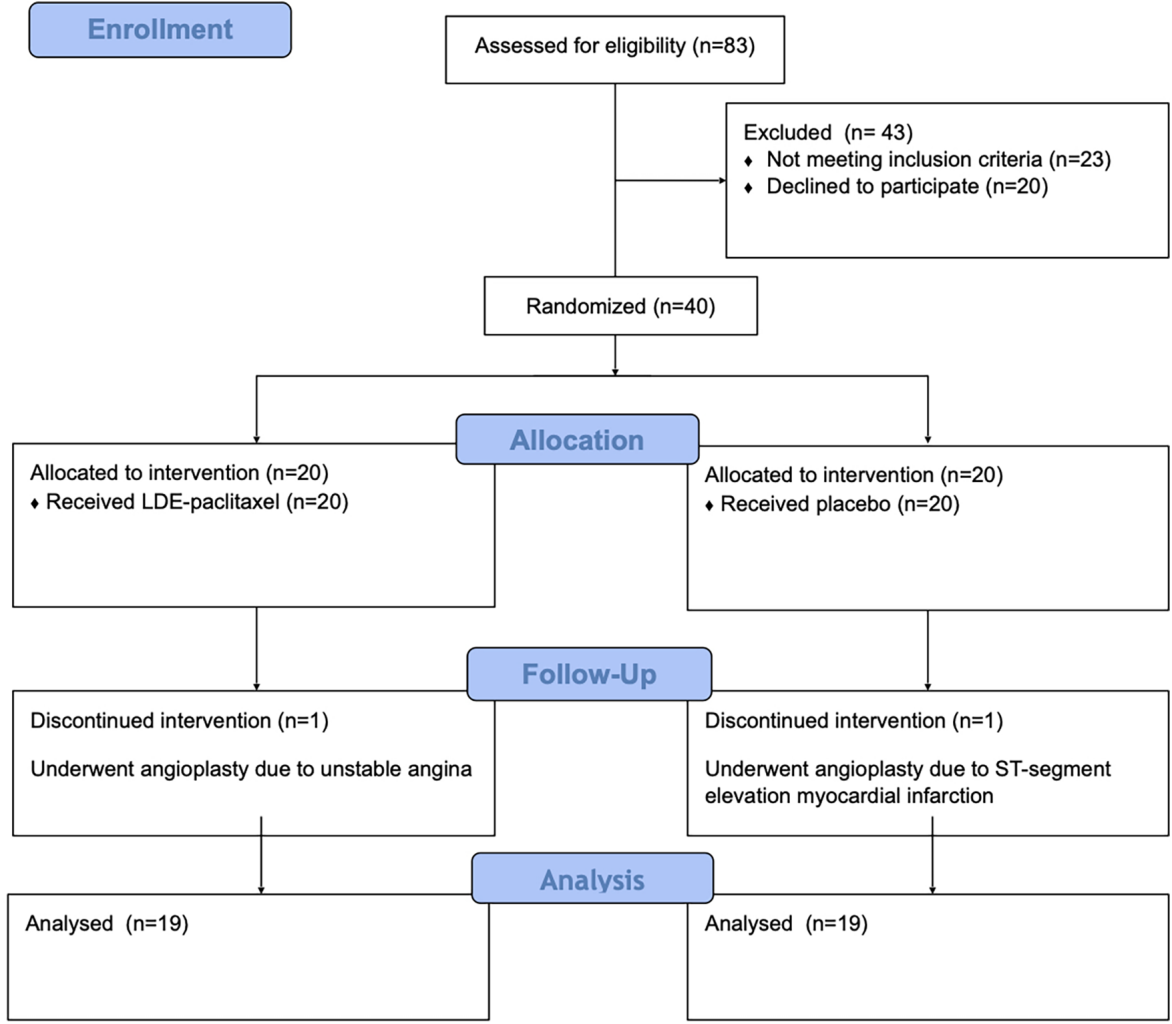
CONSORT diagram.

Between June 2019 and January 2020, we recruited 40 patients from the chronic coronary disease clinic at Heart Institute (InCor), University of Sao Paulo Medical School Hospital (Figure 1). Patients were randomly assigned in 1:1 ratio to receive either LDE-paclitaxel (paclitaxel dose: 175 mg/m^2^ body surface) or LDE alone (placebo group). In both groups, patients received intravenous treatments at three-week intervals for a total of six cycles. All patients received optimal medical therapy as determined by their attending cardiologist. The preparation of LDE particles containing paclitaxel and the infusion protocol has been previously described (12).

Patients aged 18 years or older were eligible for inclusion if they had established multivessel chronic coronary artery disease confirmed through previous coronary CTA or invasive angiography. Exclusion criteria comprised pregnancy or pregnancy risk, lactating women, a history of coronary artery bypass graft, an episode of acute coronary syndrome within the past 30 days, chronic kidney disease with creatinine levels >1.5 mg/dl, severe liver disease, hypersensitivity or allergy to paclitaxel, ongoing paclitaxel treatment for a different condition, presence of chronic infections (such as tuberculosis, HIV, Hepatitis B or C), heart failure with an ejection fraction below 40%, New York Heart Association (NYHA) class III/IV heart failure, Canadian Cardiovascular Society (CCS) class III/IV angina, chronic or acute aortic dissection, a history of cancer or chronic inflammatory disease, or current use of glucocorticoids or other immunosuppressive therapies.

The protocol was approved by the local institutional review board and ethics committee, and all participants provided written informed consent in the present study.

### Clinical data, laboratory measurement, and follow-up monitoring

All participants underwent a comprehensive medical history assessment, physical examination, medication review, and anthropometric measurements both at baseline and 4 weeks after the last visit. Blood samples were obtained after an overnight fast of 12 hours and were promptly processed for analysis during these time points. The measurements included hemoglobin, white blood cell count, platelet count, hsCRP, urea, creatinine, hepatic enzymes, LDL cholesterol, total cholesterol (TC), high-density lipoprotein cholesterol (HDL-C), triglycerides, and creatine phosphokinase (CPK) levels, utilizing automated diagnostic instruments. Additional blood samples were collected at baseline and 4 weeks after the last visit, stored, and subsequently analyzed collectively upon the study's conclusion to determine levels of IL-1β and IL-6.

Patients underwent a comprehensive medical assessment every 21 days, including physical examination, medication evaluation, and an adverse events questionnaire. Concomitantly, the following laboratory tests were performed to assess toxicity: hemoglobin, white blood cells, platelets, urea, creatinine, and hepatic enzymes. Furthermore, patients had the option to communicate with the research team at any time to report new symptoms. The evaluation of toxicity levels, in accordance with the NCI Common Terminology Criteria for Adverse Events v5.0 (CTCAE) (13), was conducted prior to the initiation of each treatment cycle. Treatment would be halted and postponed for 7 days if moderate (grade 2) adverse clinical or laboratory events were identified. Following the resolution of clinical symptoms and the normalization of laboratory parameters within safe limits, the study drug administration would recommence at a reduced dose of 75 mg/m^2^ body surface area. This dosage would be incrementally increased by 50 mg/m^2^ towards the maximum target dose in the ensuing weeks. In cases of mild (grade 1) adverse events, administration of symptomatic medications not associated with a severe interaction with paclitaxel was permitted to mitigate side effects without interrupting study drug administration.

Patients were withdrawn from the protocol if they had any severe toxicity symptoms or signs or if they experienced acute coronary syndrome or decompensated heart failure during the follow-up.

### Coronary CTA acquisition

Coronary CTA was performed using a 320-row detector scanner (GE Healthcare, Milwaukee, Wisconsin). The imaging protocol consisted of a retrospectively ECG-gated, contrast-enhanced coronary CTA, according to the Society of Cardiac Computed Tomography (SCCT) guidelines (14). Coronary CTA was performed at both baseline and within four weeks after the final intervention. Identical protocols, image settings, and contrast medium dosages were maintained for both baseline and follow-up evaluations. To achieve a resting heart rate below 65 beats per minute, intravenous metoprolol (up to 15 mg) or oral metoprolol (up to 100 mg) was administered as needed. Additionally, sublingual isosorbide dinitrate (5 mg) was administered for coronary vasodilation unless contraindicated. The following imaging and reconstruction parameters were used: a collimation of 64.000 mm × 0.625 mm, tube voltage of 120 kV, and tube current ranging from 400 to 580 milliamperes.

### Coronary plaque volume and composition

An experienced cardiac CTA reader, blinded to clinical data and randomization, assessed coronary segments using a semi-automatic 3D workstation Vitrea 2 (Vital lmages). For the quantitative assessment of coronary atherosclerotic plaques, the diameters, areas, and volume of all plaques exceeding 1 mm in thickness in all major coronary branches larger than 1.5 mm were measured according to the 17-segment model established by the Society of Cardiovascular Computed Tomography (SCCT) (14). Baseline and follow-up CTAs were assessed side by side to minimize interscan measurement variability. We measured the volume and composition of individual plaques, keeping the measured length constant between baseline and follow-up.

Plaque volume was calculated as the sum of all voxels between the inner and outer vessel contours, which were generated automatically by the software and edited manually if necessary. To determine the plaque composition, we split the plaque volume into four components using consecutive radiodensity thresholds: low attenuation < 30HU (Hounsfield Units), fibro-fatty 30–150HU, fibrotic 151–350, and dense calcified > 350HU (15–17). The total plaque volume, measured in mm^3^, was calculated by summing the volumes of all previously specified plaque subcomponents.

For the assessment of atherosclerotic plaque progression during the follow-up and comparison between groups, the mean values of total plaque volume and volumes of each plaque subcomponent were calculated per patient at baseline and follow-up. Plaque volume change was defined as a follow-up—baseline mean volume. Plaque progression and regression were defined as any increase or decrease in plaque volume, respectively. The percentage plaque volume was calculated as the difference between the means of the follow-up plaque volume minus the baseline plaque volume, divided by the mean of plaque volume at baseline, and then multiplied by 100%: [(Follow-up plaque volume - Baseline plaque volume)/Baseline plaque volume] × 100%. In addition, in our analysis at the patient level, we evaluated segments with stenting, chronic occlusion, or issues with image reconstruction and excluded from plaque progression final analysis.

### Statistical analysis

Baseline characteristics were compared between groups. Distributions of continuous variables were analyzed for normality using the Kolmogorov–Smirnov test. Continuous data were expressed as means ± SD or median (minimum, maximum or interquartile ranges) according to distribution. Between-group differences in normally distributed variables were analyzed using an unpaired *t*-test. For non-Gaussian distributed variables, the Mann–Whitney *U*-test was used. To compare baseline and follow-up data within the same group, a paired t-test or Wilcoxon test was used according to distribution. Categorical data were summarized as percentages and analyzed with the χ^2^ or Fisher exact test. We also performed a repeated measure 2-way mixed analysis of variance to determine if an interaction effect exists between the treatment group (placebo vs. LDE-paclitaxel) and time (baseline vs. follow-up) for the outcome variables.

All analyses from data tables were performed with SAS version 9.4 (SAS Institute Inc., Cary, NC) and all analyses from figures were performed with GraphPad Prism version 9.0 (GraphPad Software, San Diego, CA). For sample-size calculation, software STATA version 14.0 was used. A 2-tailed probability value <0.05 was calculated to indicate statistical significance.

## Results

### Baseline characteristics

The median study follow-up was 176 days (minimum: 147, maximum: 236). The characteristics of participants at baseline were similar in the LDE-paclitaxel and placebo groups (Table 1). Of the 40 patients randomized, 38 completed the study. One patient in each arm had an acute coronary syndrome during the follow-up, and according to protocol, treatment was interrupted, and these patients were excluded from the final analysis.

**Table 1:** Characteristics of the patients at baseline.

	LDE-paclitaxel (*n* = 19)	Placebo (*n* = 19)	*p*
Mean age ± SD, year	65.4 ± 8.9	64.3 ± 7.1	0.66
Female sex, *N* (%)	2 (10.5)	6 (31.6)	0.23
Mean BMI ± SD, kg/m^2^	29.1 ± 3.7	28.5 ± 3.6	0.65
Diabetes, *N* (%)	9 (47.4)	13 (68.4)	0.19
Smoking, *N* (%)	11 (57.9)	13 (68.4)	0.50
Hypertension, *N* (%)	17 (89.5)	18 (94.7)	1.0
CKD, *N* (%)^a^	3 (15.8)	4 (21.1)	1.0
Previous MI, *N* (%)	11 (57.9)	8 (42.1)	0.33
Previous PCI, *N* (%)	10 (52.6)	8 (42.1)	0.52
Concomitant medications, *N* (%)			
Statin	19 (100)	18 (94.7)	1.0
Aspirin	19 (100)	18 (94.7)	1.0
Clopidogrel	5 (26.3)	6 (31.6)	0.72
Follow-up days, [minimum, maximum]	166 [147–229]	189 [148–236]	0.17
Current CCS angina class I	6 (31.6)	3 (15.8)	0.45
Mean lipid levels ± SD, mg/dl			
Total cholesterol	132 ± 42	143 ± 27	0.31
LDL cholesterol	69 ± 30	78 ± 19	0.31
HDL cholesterol	38 ± 10	42 ± 10	0.34
Triglycerides	121 ± 72	124 ± 73	0.92

SD, denotes standard deviation; BMI, body mass index; CKD, chronic kidney disease; MI, myocardial infarction; PCI, percutaneous coronary intervention; CCS, Canadian Cardiovascular Society.

^a^ eGFR < 60 ml/min/1.73 m^2^.

The mean age was 65 years, 21% were women, and half of patients had a history of MI. Approximately two-thirds had a history of smoking. Nearly 60% of the population had diabetes, and more than 90% had hypertension. At baseline, more than 90% of participants in both groups were taking statins and aspirin, and 29% were on dual antiplatelet therapy with clopidogrel. More than two-thirds of patients were asymptomatic at baseline, with only 9 patients presenting with symptoms of angina CCS 1. The mean (±SD) baseline total cholesterol was 138 ± 35 mg/dl, and LDL-C was 73 ± 25 mg/dl, with no statistical difference between groups (Table 1).

### Inflammatory and lipid biomarkers change at follow-up

**Figure 2:**
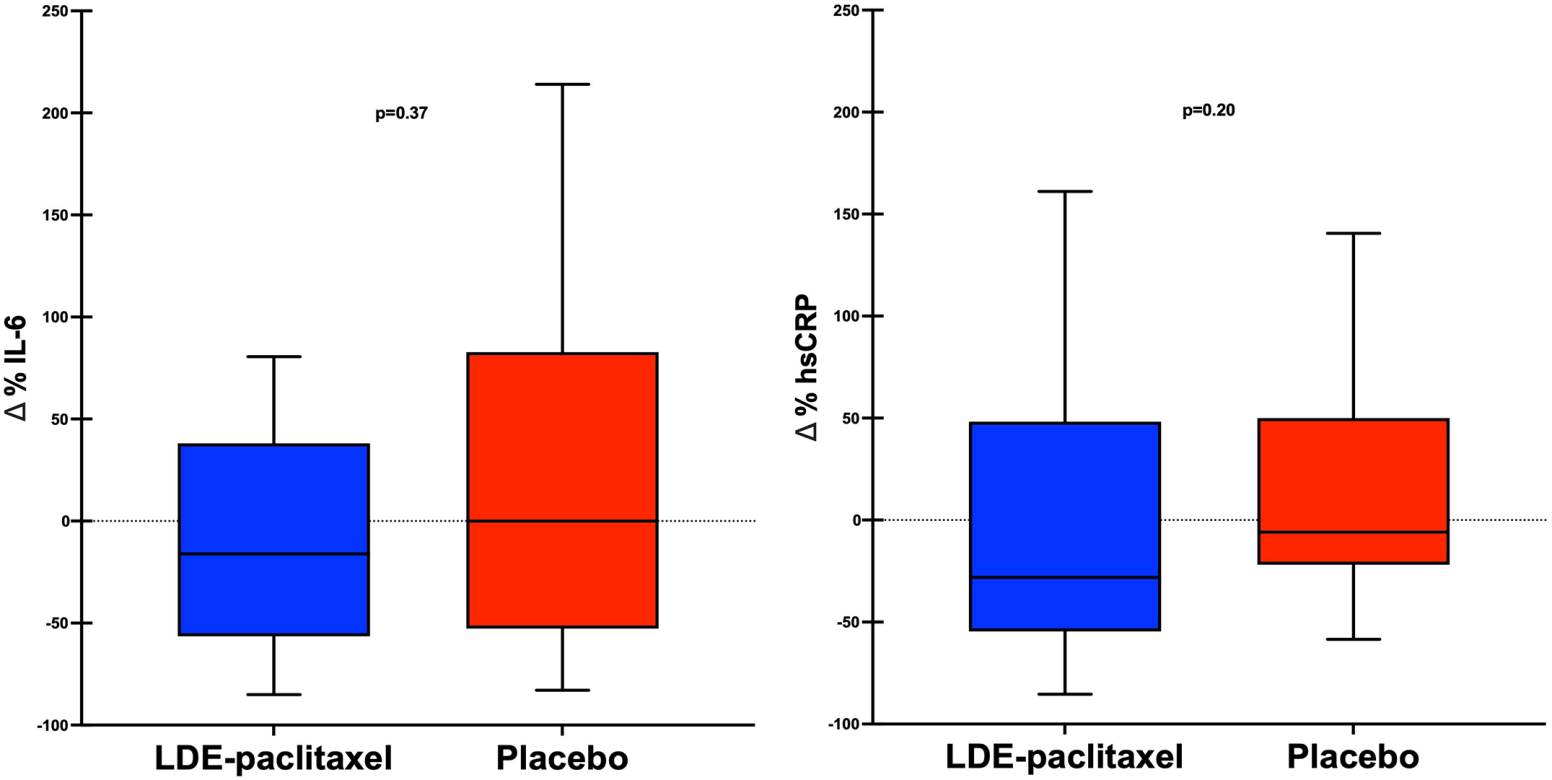
Comparison of percentual variation (*Δ*%) in IL-6 and hsCRP levels during the follow-up.

At baseline and follow-up, there were no statistical differences in inflammatory biomarker levels in both groups (Table 2). However, we observed a more pronounced reduction in hsCRP and IL-6 levels in the LDE-paclitaxel group compared to the placebo group (−28.1% vs. −5.9%, *p* = 0.20 for hsCRP and −16.1% vs. 0%, *p* = 0.37 for IL-6) (Figure 2). Furthermore, there were no statistically significant changes in lipid levels in either of the groups during the follow-up period (Supplementary Table S1).

**Table 2:** Baseline, follow-up, and changes in inflammatory biomarkers in the placebo and treatment groups.

	Baseline			Placebo			LDE-paclitaxel		
	Placebo (*n* = 19)	LDE-paclitaxel (*n* = 19)	*p*-value*	Follow-up (*n* = 19)	Change (%) from baseline	*p*-value^†^	Follow-up (*n* = 19)	Change (%) from baseline	*p*-value^‡^
hsCRP (mg/L)	1.79 (0.55–3.35)	1.25 (0.57–2.96)	0.60	1.59 (0.66–3.71)	−5.9 (−21.8:+50.0)	0.56	1.12 (0.41–1.59)	−28.1 (−54.6:+48.2)	0.15
IL-6(pg/ml)	4.33 (2.59–13.8)	5.31 (1.56–8.56)	0.59	5.31 (2.05–13.8)	0 (−52.7:+82.8)	0.90	3.47 (1.56–7.55)	−16.1 (−56.5:+38.0)	0.14
IL-1β (pg/ml)	0.44 (0.37–0.89)	0.44 (0.37–0.44)	0.12	0.44 (0.37–0.70)	0 (−15.9:+23.3)	0.83	0.44 (0.37–0.52)	+18.2 (0:+18.9)	0.19

Values are median (IQR).

* *p*-value comparing baseline values between the 2 groups. *p*-values comparing baseline and follow-up values in the ^†^control group and the ^‡^treatment group.

### Plaque volume changes at follow-up

For the quantitative assessment of coronary atherosclerotic plaques, three patients were excluded from this analysis due to defects in the reconstruction of the follow-up coronary CTA images. A total of 35 patients, 18 in the LDE-paclitaxel group and 17 in the placebo group, were analyzed for assessment of plaque volume changes after the intervention. In our analysis, we examined a total of 199 plaques, with 107 in the LDE-paclitaxel group and 92 in the placebo group.

The baseline and follow-up median values and changes in coronary CTA plaque parameters for both the LDE-paclitaxel and placebo groups are summarized in Table 3. The median volume of low attenuation plaques was 39.6 (29.4–57.4) mm^3^, and it is noteworthy that no significant differences were observed between the two groups in this or any other plaque subcomponents at baseline. During the follow-up, there were no statistically significant changes in the volume of low attenuation plaques in the treatment or placebo group (*p* = 0.37 and *p* = 0.82, respectively). Furthermore, no other changes were observed in the coronary CTA plaque parameters of interest in either group.

**Table 3:** Baseline, follow-up, and changes in CTA plaque parameters in the placebo and treatment groups.

Plaque volume	Baseline			Placebo			LDE-paclitaxel		
	Placebo (*N* = 92)	LDE-paclitaxel (*N* = 107)	*p*-value*	Follow-up (*n* = 92)	Change (%) from baseline	*p*-value^†^	Follow-up (*n* = 107)	Change (%) from baseline	*p*-value^‡^
Low-attenuation (mm^3^)	39.6 (29.4–57.4)	39.7 (32.7–58.1)	0.96	40.0 (29.8–57.1)	0.5 (−22.8:+21.0)	0.82	39.0 (27.5–54.5)	−1.8 (−19.0:+15.5)	0.37
Fibro-fatty (mm^3^)	93.1 (73.7–108.9)	86.4 (56.5–132.4)	0.73	85.6 (74.0–122.0)	−1.2 (−12.3:+10.7)	0.85	81.2 (60.5–121.6)	−2.0 (−10.1:+8.3)	0.35
Fibrotic (mm^3^)	57.3 (41.7–63.2)	54.1 (31.0–75.3)	0.93	54.9 (39.8–67.1)	−5.0 (−10.5:+1.7)	0.11	53.3 (31.1–79.4)	4.4 (−6.3:+9.9)	0.28
Dense-calcified (mm^3^)	17.2 (7.8–25.2)	12.4 (6.2–21.3)	0.70	11.7 (7.4–25.8)	−7.5 (−22.5:+21.7)	0.23	12.3 (6.4–24.8)	−17.3 (−26.4:+33.8)	0.14
Total (mm^3^)	206.9 (170.0–252.8)	189.6 (142.5–267.1)	0.71	196.9 (175.7–325.0)	−6.1 (−10.4:+4.8)	0.40	186.7 (137.1–264.1)	−0.2 (−8.1:+11.0)	0.77

Values expressed as median (IQR). *N* refers to number of plaques.

* *p*-value comparing baseline values between the 2 groups. *p*-values comparing baseline and follow-up values in the ^†^control group and the ^‡^treatment group.

### Adverse events

During the follow-up, two patients had a major adverse cardiovascular event, and according to protocol, treatment was interrupted, and these patients were excluded from the final analysis. One patient in the LDE-paclitaxel group was admitted with chest pain one week after the first dose of the medication, with no changes in troponin levels or ECG. The hypothesis of unstable angina was considered, and the patient underwent coronary angiography, which showed no significant differences compared to the previous exam, with an approximate 70% stenosis of the left anterior descending artery (LAD) and angioplasty of this vessel was performed by the attending interventional cardiologist. The other patient, from the placebo group, experienced typical chest pain three weeks after randomization and was diagnosed with ST-segment elevation myocardial infarction (STEMI) and subsequently underwent LAD angioplasty. Noteworthy, no patients exhibited worsening or new symptoms of angina or heart failure during the follow-up.

In none of the patients treated with LDE-paclitaxel, moderate or severe toxicities were observed during the follow-up (Table 4). Common moderate or severe adverse events associated with conventional paclitaxel are myelosuppression, nausea, alopecia, arthralgia, myalgia, and peripheral neuropathy, among others less frequent did not appear. In addition, there were no reports of serious adverse infection events in either group. During the follow-up, there were no significant changes in creatinine, urea, AST, ALT, red blood cell, white blood cell, platelet, or CPK levels when compared to baseline (Table 5). Furthermore, no significant changes in these parameters were observed after each treatment cycle in both groups (Supplementary Figure S1–S7). A non-significant trend was observed in AST and ALT levels in the LDE-paclitaxel group; however, there were no significant changes in final levels of AST and ALT (*p* = 0.31 and *p* = 0.19, respectively), and those levels remained below three times the upper limit value.

**Table 4:** Investigator-reported adverse clinical events*.

	LDE-paclitaxel (*n* = 19)	Placebo (*n* = 19)	*p*
Grade 1 toxicity^†^			
Alopecia, *N* (%)	1 (5.3)	0	0.31
Arthralgia, *N* (%)	2 (10.5)	5 (26.3)	0.21
Dermatitis, *N* (%)	2 (10.5)	2 (10.5)	1.0
Diarrhea, *N* (%)	5 (26.3)	2 (10.5)	0.21
Dyspnea, *N* (%)	4 (21.1)	3 (15.8)	0.68
Local pain, *N* (%)	1 (5.3)	1 (5.3)	1.0
Fatigue, *N* (%)	1 (5.3)	3 (15.8)	0.29
Myalgia, *N* (%)	0	0	–
Mucositis, *N* (%)	2 (10.5)	5 (26.3)	0.21
Nausea, *N* (%)	2 (10.5)	2 (10.5)	1.0
Neurotoxicity, *N* (%)	3 (15.8)	4 (21.1)	0.68
Pruritus, *N* (%)	3 (15.8)	2(10.5)	0.63

* These events were defined in the trial protocol and queried at each visit.

^†^ Toxicity was classified from grade 1 (mild; asymptomatic or mild symptoms; clinical or diagnostic observations only; intervention not indicated) to grade 5 (death related to adverse event). The data represent the number of patients who presented grade 1 toxicity only because no grade 2–5 toxicities were observed.

**Table 5:** Baseline and follow-up levels of renal, liver, and hematologic safety parameters in the placebo and treatment groups.

	LDE-paclitaxel (*n* = 19)			Placebo (*n* = 19)		
	Baseline	Follow-up	*p*	Baseline	Follow-up	*p*
Creatinine, mg/dl	0.97 ± 0.1	1.02 ± 0.1	0.07	0.98 ± 0.2	1.01 ± 0.2	0.26
Urea, mg/dl	37 ± 10	36 ± 11	0.54	38 ± 11	36 ± 11	0.24
AST, U/L	25 ± 13	44 ± 88	0.31	25 ± 9	25 ± 11	0.85
ALT, U/L	40 ± 22	56 ± 65	0.19	34 ± 14	35 ± 13	0.77
CPK, U/L	135 ± 61	152 ± 75	0.18	150 ± 97	146 ± 106	0.84
Hemoglobin, g/dl	14.6 ± 1.2	14.7 ± 1.1	0.52	13.9 ± 1.6	13.9 ± 1.4	0.91
WBC, mm^3^	7,389 ± 1,639	7,474 ± 2,132	0.81	7,805 ± 1,440	7,591 ± 1,833	0.69
Platelets, mm^3^	244,157 ± 57,782	242,105 ± 61,754	0.83	221,631 ± 51,718	230,789 ± 58,870	0.82

Values expressed as mean ± SD.

AST, denotes aspartate aminotransferase; ALT, alanine aminotransferase; CPK, creatine phosphokinase; and WBC, white blood cells.

## Discussion

In this randomized, placebo-controlled pilot clinical trial, LDE associated with paclitaxel showed absence of clinical and laboratory toxicity in patients with multivessel chronic coronary artery disease and optimized medical therapy.

While no statistically significant reduction in inflammatory biomarkers was observed during the follow-up, interesting trends were noted. In the group treated with LDE-paclitaxel, the median IL-6 values decreased by 16%, whereas in the placebo group IL-6 was unchanged as compared to baseline (*p* = 0.37). IL-6 is known to play a crucial role in atherosclerotic disease (18). In this respect, for each increase of 1 standard deviation in IL-6 levels, the risk of MI or cardiovascular death increased by 25% (19), and for this reason, the benefit of Ziltivekimab, a direct inhibitor of IL-6, is being evaluated in an ongoing trial (20). In addition, the levels of hsCRP also decreased more prominently in the LDE-paclitaxel group compared to the placebo group (−28.1% vs. −5.9%, *p* = 0.20). It is well known that hsCRP acts downstream of the inflammatory cascade, with IL-6 being of fundamental importance in its expression and function (21, 22). Unexpectedly, IL-1β levels increased in the LDE-paclitaxel group (+18.2%), while they remained unchanged in the placebo group (*p* = 0.37). This unexpected pattern may be due to the variability and very low systemic levels of IL-1β, which makes its accurate measure a challenge. Moreover, most of the prospective observational data concerning IL-1β and high cardiovascular risk has been derived from studies involving the IL-1 receptor antagonist, which exhibits a strong correlation with IL-1β activity and provides more precise measurements (23). Our data suggest that the intervention with LDE-paclitaxel may have some effect on inflammatory biomarkers associated with cardiovascular disease, particularly IL-6, and hsCRP. Our pilot study had limited statistical power to detect differences in inflammatory biomarker levels. Further research is needed to explore the potential modulation of these important pathways in atherosclerosis by LDE-paclitaxel.

The association of non-calcified plaques with higher cardiovascular risk is well-established, and lipid-lowering therapies have shown regression or a slowing of non-calcified plaque progression (24, 25). However, similar findings with immunomodulatory therapies remain scarce. A pilot study with Canakinumab, an IL-1β inhibitor, did not convey regression in superficial femoral arteries plaque volume after 12 months of treatment assessed by magnetic resonance imaging (26). In a secondary prevention cohort, treatment with colchicine at 0.5 mg/day dose resulted in a 41% reduction in low-attenuation plaque volume after 12 months of follow-up (11). It is known that plaque components with lower radiodensity (<30 UH) typically correspond to the lipid core, and when this volume accounts for more than 4% of the total vessel volume, the risk of MI is 5-fold higher (27).

Paclitaxel enhances tubulin polymerization and stabilizes cell microtubules, leading to the inhibition of mitotic apparatus formation and cell cycle arrest in the G2/M phase (28, 29). In our study, we observed stability in low-attenuation plaque volume after intervention in both groups. Several limitations of our study may account for the absence of significant changes in plaque volume. Firstly, our follow-up was approximately 6 months, whereas most studies assessing plaque progression had at least 12 months of follow-up (11, 30). However, a shorter follow-up period might be justified to ensure patient safety, considering this was the first randomized study to test systemic use of LDE-paclitaxel in patients with coronary artery disease. Secondly, despite a similar mean value of low-attenuation plaque volume in our study compared to the Vaidya et al. (11) (45.1 mm^3^ vs. 39.0 mm^3^), the standard deviation of our sample was twice as high (24.6 mm^3^ vs. 10.9 mm^3^). Consequently, our statistical power was insufficient to detect a significant difference in low-attenuation plaque volume between the groups. Finally, changes in plaque characteristics represent a more advanced phase in atherogenesis compared to the early changes observed in inflammatory biomarkers, occurring later in patients with chronic coronary artery disease.

Unlike colchicine, which is taken orally once daily, LDE-paclitaxel requires intravenous administration every three weeks, which is perhaps not practical for long-term therapy. In patients with high inflammatory risk and plaque burden, there is a need for a potent and rapid pharmacological action, and we deem LDE-paclitaxel may have room in this scenario. In previous studies, reduction in plaque progression was achieved with anti-inflammatory therapy in patients with acute coronary syndrome in the last 30 days: at this stage, systemic and local inflammation at the plaque site is heightened (11, 31). Therefore, further studies evaluating the effect of LDE-paclitaxel on atherosclerotic plaque progression and inflammatory biomarkers should consider to include patients with recent acute myocardial infarction.

In our study, it was observed absence of significant clinical or laboratory adverse events related to paclitaxel in the intervention group. This finding reinforces the safety of LDE-paclitaxel in non-oncological patients, which has already been observed in a non-randomized observational study with only 8 patients (12). The development of new formulations using nanotechnology is already a reality in the oncology therapeutic field (32). The exact mechanism by which the LDE formulations exhibit a markedly lower incidence of toxicity is not fully understood. We hypothesize that several factors may contribute to this phenomenon for LDE formulation: a unique biodistribution pattern created by the carrier, which accumulates in atherosclerotic lesions and cancer cells, and drug encapsulation, which reduces exposure to normal tissues while extending the drug's half-life (8, 33).

In conclusion, treatment with LDE-paclitaxel showed tolerance and safety in a small cohort of patients with multivessel chronic coronary artery disease. The results also suggest that LDE-paclitaxel might attenuate the expression of IL-6, the key inflammatory mediator of cardiovascular disease. Thus, our study may encourage and furnish valuable data for the design of future clinical research on LDE-paclitaxel to investigate the effects of this formulation on the modulation of the inflammatory pathways involved in atherosclerotic cardiovascular disease.

## Data Availability

The raw data supporting the conclusions of this article will be made available by the authors, without undue reservation.
