# Supplementary material for: Safety and possible anti-inflammatory effect of paclitaxel associated with LDL-like nanoparticles (LDE) in patients with chronic coronary artery disease: a double-blind, placebo-controlled pilot study
Source: Front Cardiovasc Med. 2024 Feb 21;11:1342832. doi: 10.3389/fcvm.2024.1342832 (PMC10915057; doi:10.3389/fcvm.2024.1342832)
Supplement: Supplementary file 1 [file Table1.docx]

| **Table S1.** Baseline and Follow-Up Lipid Parameters in the Treatment and Placebo Groups | | | | | | |
| --- | --- | --- | --- | --- | --- | --- |
|  | LDE-paclitaxel (n=19) | |  | Placebo (n=19) | |  |
|  | **Baseline** | **Follow-up** | **p** | **Baseline** | **Follow-up** | **p** |
| Total cholesterol (mg/dL) | 132±42 | 135±36 | 0.67 | 143±27 | 139±31 | 0.47 |
| LDL cholesterol (mg/dL) | 69±30 | 72±25 | 0.66 | 78±19 | 75±26 | 0.65 |
| HDL cholesterol (mg/dL) | 38±10 | 39±10 | 0.61 | 42±10 | 42±10 | 0.75 |
| Triglycerides (mg/dL) | 121±72 | 121±67 | 0.98 | 124±73 | 107±50 | 0.27 |
| Values are mean ± SD. | | | | | | |
